# Characterization of the Gut Microbiota in the Red Panda (*Ailurus fulgens*)

**DOI:** 10.1371/journal.pone.0087885

**Published:** 2014-02-03

**Authors:** Fanli Kong, Jiangchao Zhao, Shushu Han, Bo Zeng, Jiandong Yang, Xiaohui Si, Benqing Yang, Mingyao Yang, Huailiang Xu, Ying Li

**Affiliations:** 1 Institute of Animal Genetics and Breeding, Sichuan Agricultural University, Chengdu, Sichuan, China; 2 Department of Pediatrics and Communicable Disease, University of Michigan, Ann Arbor, Michigan, United States of America; 3 Sichuan Agricultural University, College of Animal Science and Technology, Ya'an, Sichuan, China; 4 Sichuan Fengtongzhai National Nature Reserve, Baoxing, Sichuan, China; Loyola University Medical Center, United States of America

## Abstract

The red panda is the only living species of the genus *Ailurus*. Like giant pandas, red pandas are also highly specialized to feed mainly on highly fibrous bamboo. Although several studies have focused on the gut microbiota in the giant panda, little is known about the gut microbiota of the red panda. In this study, we characterized the fecal microbiota from both wild (n = 16) and captive (n = 6) red pandas using a pyrosequecing based approach targeting the V1-V3 hypervariable regions of the 16S rRNA gene. Distinct bacterial communities were observed between the two groups based on both membership and structure. Wild red pandas maintained significantly higher community diversity, richness and evenness than captive red pandas, the communities of which were skewed and dominated by taxa associated with Firmicutes. Phylogenetic analysis of the top 50 OTUs revealed that 10 of them were related to known cellulose degraders. To the best of our knowledge, this is the first study of the gut microbiota of the red panda. Our data suggest that, similar to the giant panda, the gut microbiota in the red panda might also play important roles in the digestion of bamboo.

## Introduction

Red pandas (*Ailurus fulgens*) are attractive animals endemic to the temperate forests of the Himalayas, ranging from the foothills of western Nepal to the southwest of China [1]. The red panda is the only living species of the genus *Ailurus* and the family *Ailuridae* [2]. Although protected by national laws in their range countries, the population of the red panda continues to decline and has been classified as vulnerable by the International Union for Conservation of Nature (IUCN) due to factors such as habitat loss, hunting, and inbreeding depression [3]. Given the many threats that this endangered species faces, many of them have been raised in zoos for better care and protection. As of 2006, about 800 red pandas had been listed by the international studbook as living in zoos or parks worldwide [4].

Whereas red pandas eat a large variety of foods including birds, flowers, eggs, berries, mushrooms, and maple and mulberry leaves, their major food source is bamboo, which occupies over 90% of their diet [5]. However, like the giant panda, the red panda also has a short and relatively simple digestive tract typical of other carnivores and does not process bamboo well, especially the cellulose components of the plant cell walls [6], [7]. It has been well established that the gut microbiota plays an essential role in nutrition uptake, energy harvest, food digestion and vitamin synthesis [8], [9] in humans and other animals. Culture-independent metagenomic analysis has revealed the presence of cellulose degraders in the gut microbiota of the giant panda [10], [11]. However, the composition, structure and role of the gut microbiota in the red panda remain largely unknown. In this study, we characterized the fecal microbiota from both wild and captive red pandas. We hypothesize that: i) the gut microbiota of wild and captive red pandas differs in membership and structure; and ii) the gut microbiota of red pandas consists of members related to cellulose digestion.

## Results

### Sequencing depth and alpha diversities

Twenty two fecal samples were collected from wild (n = 16) and captive (n = 6) red pandas. DNA was extracted from these samples and was subjected to bar-coded pyrosequencing of the V1–V3 region of the 16S rRNA gene. Sequences were processed and analyzed using mothur v1.31 [12]. Sequencing error and chimeras were detected and removed by using the default settings in mothur. After removing the low quality reads and chimeras, 63,622 high quality reads remained with an average of 2892 reads per sample, ranging from 1365 to 5929. These sequences, with median (interquartile range) length of 248 (232–257) bp, were assigned to 477 operational taxonomic units (OTUs) based on 97% similarity. The average Good's coverage was 99.1%±0.6% (mean ± SD, range  = 97.2–99.9%, Table S1). For the downstream alpha and beta diversity analyses, sequence number was normalized to 1300 by randomly subsampling to standardize sampling effort. The subsampling of the sequences still yielded sufficient resolution of bacterial communities, as suggested by an average Good's coverage of 98.6%±0.8% (mean ± SD, Table S1) and by rarefaction curve analysis (Figure S1).

**Figure 1:**
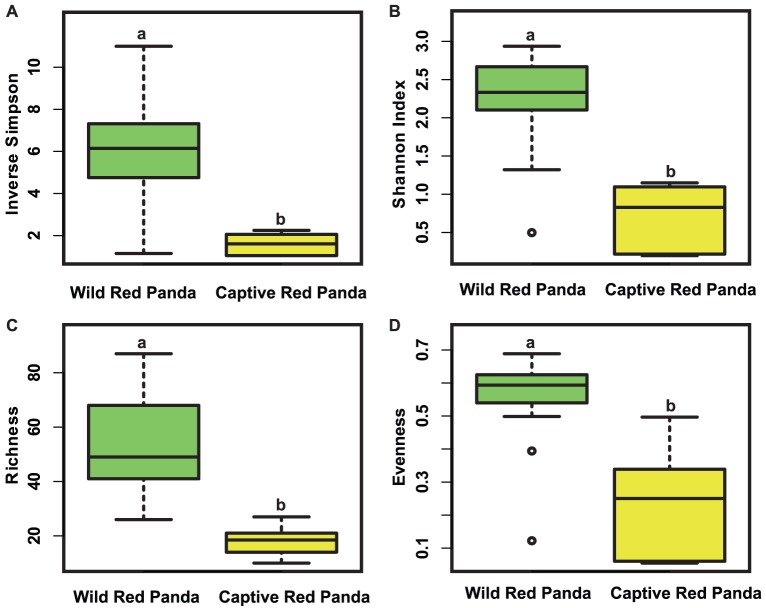
Comparison of community alpha diversities between the wild and captive red pandas. Diversity was measured by inverse Simpson (A) and Shannon index (B); Richness (C) and evenness (D) were measured by the number of observed OTUs and Shannon Evenness index, respectively. The top and bottom boundaries of each box indicate the 75^th^ and 25^th^ quartile valudes, respectively. The black lines within each box represent the median values. Different lowercase letters above the boxplots indicate significant differences in alpha diversities between wild and captive pandas (P<0.001, Mann Whitney test).

Bacterial community diversity was measured by both Shannon index and inverse Simpson index. Both indices were significantly higher in the wild than in the captive red pandas (Figure 1A and B, Mann Whitney test, P<0.001). Consistently, the community richness (total number of observed OTUs) and evenness (Shannon evenness) in the wild red panda were significantly higher than those in the captive red panda (Figure 1C and D, Mann Whitney test, P<0.001).

### Different community membership and structures

**Figure 2:**
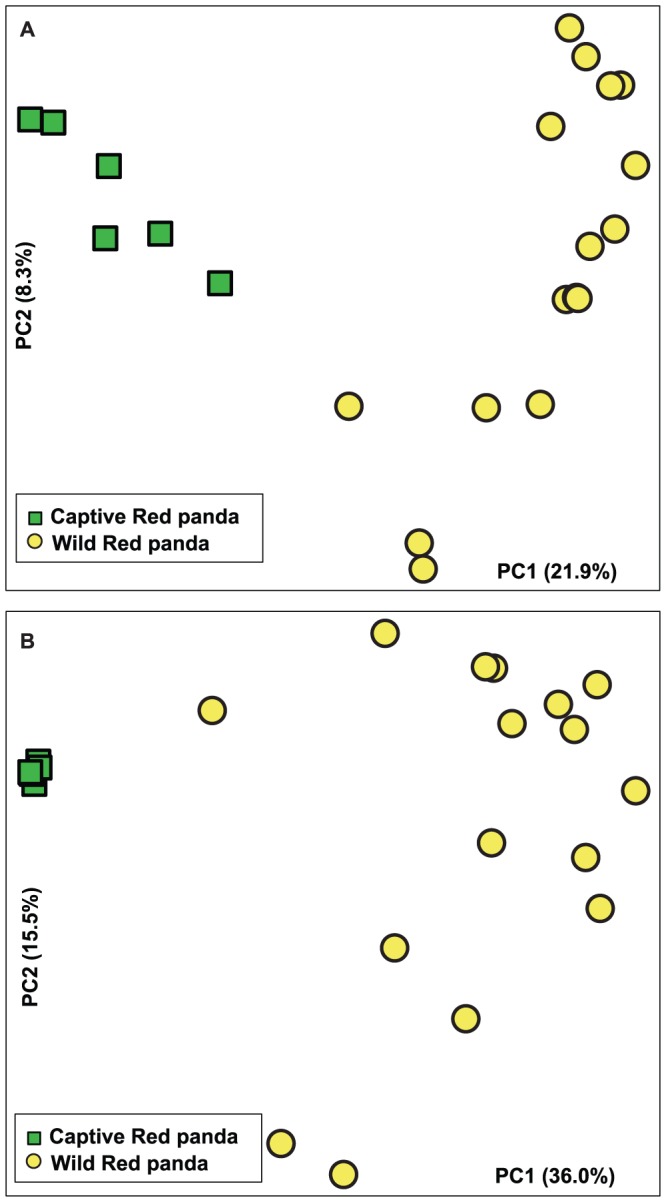
Principal coordinate analysis of the community membership (A) and structure (B) using Jaccard and Theta YC distances, respectively. Green squares and yellow circles represent captive and wild red panda bacterial communities, respectively. Distances between symbols on the ordination plot reflect relative dissimilarities in community memberships or structures.

After comparing the alpha diversities between wild and captive red pandas, we next examined the beta diversity measures between wild and captive red pandas. We first calculated the Jaccard index to estimate the dissimilarities in community membership. Principal coordinate analysis (PCoA) was applied to visualize the Jaccard distances and showed that the captive and wild red panda harbor distinct bacterial taxa. Based on membership, bacterial communities from captive red pandas clustered together and separated from those from wild red pandas along principal coordinate axis 1 (PC1), which explained the largest amount of variation (21.9%, Figure 2A). This result is consistent with the alpha diversity analysis, where wild red pandas were found to possess a significantly higher number of OTUs than captive red pandas. Analysis of molecular variance (AMOVA, P<0.001) [13] revealed the differences in community membership between wild and captive red pandas were statistically significant.

**Figure 3:**
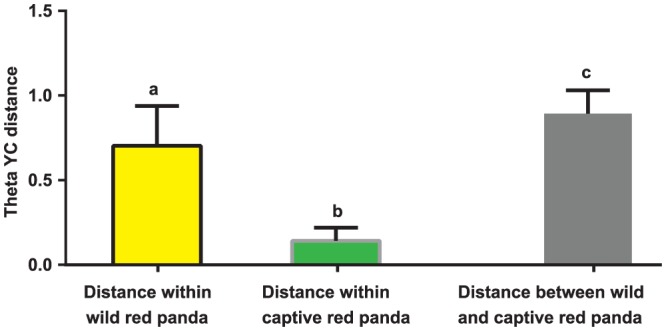
Variation of the Theta YC distances within and between the wild and captive red panda. The average Theta YC distance within the wild red panda group is significantly greater than that within the captive red panda group and smaller than the distance between the two groups as indicated by different lowercase letters (P<0.001, Kruskal-Wallis test with Dunn's correction).

To examine the dissimilarity between community structures we measured the Yue and Clayton (Theta YC) distances [14], which takes into account both membership and relative abundance. Similar to the membership-based analysis, the Theta YC PCoA plot showed that the captive red panda bacterial communities clustered tightly, and were separated from the wild red panda communities along PC1 (36.0% variation explained, Figure 2B). Interestingly, the variation within the wild red panda communities was significantly higher than that within the captive red panda communities (P<0.001, Kruskal-Wallis test with Dunn's correction, Figure 3).

### Bacterial OTUs differentially represented between wild and captive red pandas

**Figure 4:**
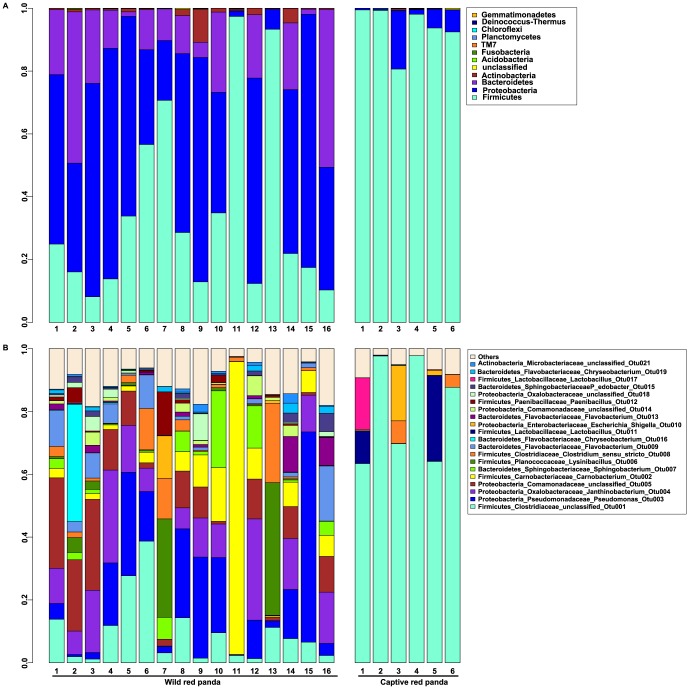
Relative abundance of OTUs at the phylum (A) and genus (B) level in the fecal microbiota from wild and captive red pandas.

The distribution of OTUs at the phylum level in wild and captive red pandas is illustrated in Figure 4A. Captive red pandas were characterized by skewed bacterial communities primarily dominated by Firmicutes, while wild red pandas possessed communities with members more evenly distributed amongst Firmicutes, Proteobacteria and Bacterioidetes. At the genus level, similar patterns were observed. Captive red pandas were dominated by an OTU associated with unclassified *Clostriaceae*. In contrast, a more diverse set of OTUs was detected in wild red pandas (Figure 4B).

**Figure 5:**
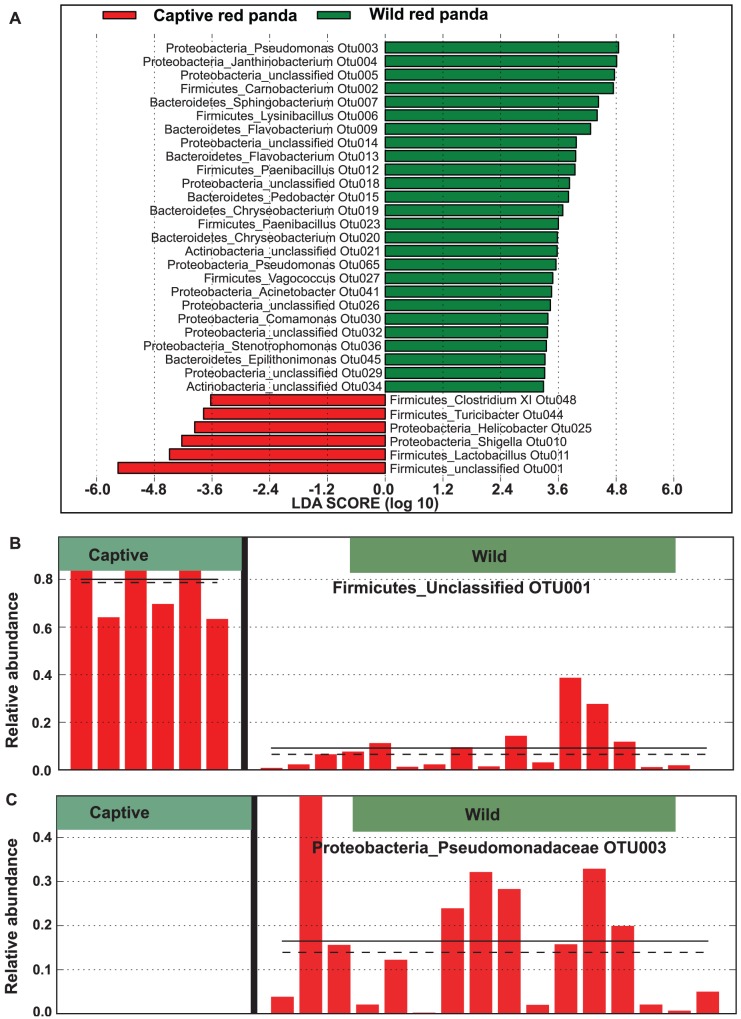
OTUs differentially represented between wild and captive red pandas identified by linear discriminant analysis coupled with effect size (LEfSe). A. Histogram showing OTUs that are more abundant in wild (green color) or captive (red color) red pandas ranked by effect size. The distribution of the most differentially distributed OTUs: OTU001 (more abundant in captive red pandas) and OTU003 (more abundant in wild red pandas) were illustrated in B and C, respectively.

To identify specific OTUs that were differentially distributed between wild and captive red pandas, we performed linear discriminant analysis effect size (LEfSe) [15], a robust tool that focuses not only on statistical significance but also biological relevance. A total of 32 OTUs were significantly differentially represented between the two groups, with 26 more abundant in wild red pandas and 6 more abundant in captive red pandas (Figure 5A). The distributions of representative OTUs from each category are illustrated in Figure 5B and C.

### Bacterial OTUs related to cellulose degradation

Although phylogenetically divergent from the giant panda, the red panda has a similar bamboo-specialized diet. Therefore, we hypothesize that members of the bacterial communities from red pandas also have the ability to degrade cellulose, as identified in communities from giant pandas [11]. We then analyzed the phylogenetic relationship between the top 50 OTUs (97% of the total sequences) detected in this study with 71 known cellulose degraders (GenBank accession number shown in table S2) and the 85 OTUs identified from giant pandas (shown in Table S3) [11]. Although selected from the pooled sequences, the top 50 OTUs were also the most abundant OTUs within each individual group of red pandas.

**Figure 6:**
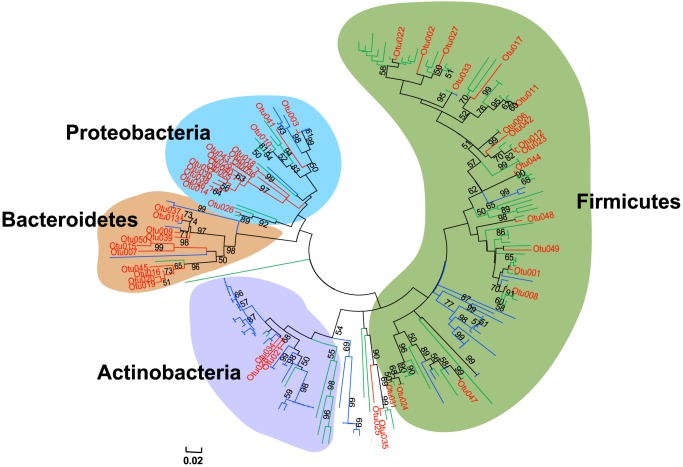
Neighbor-joining tree showing the phylogenetic relationship among the top 50 OTUs in this study (red) with known cellulose degraders (blue) and OTUs identified in the giant panda (green). Different phyla were shaded by different colors: green, Firmicutes; purple, Actinobacteria; orange, Bacteroidetes; blue, Proteobacteria. All bootstrap values > 50% were shown on the tree.

The neighbor-joining phylogenetic tree shows that, among the top 50 OTUs, the majority (18 OTUs) belong to the phylum Firmicutes (Figure 6). The Kimura 2-parameter distance [16] was used to define phylogenetic relationships between the top 50 OTUs in our study and those reported by others. In total, 10 OTUs were closely related to known cellulose degraders with a pairwise Kimura 2-parameter distance <0.03. A total of 16 OTUs matched OTUs reported by Zhu et al [11] from giant pandas (pairwise Kimura 2-parameter distance <0.03).

## Discussion

Several studies have focused on the gut microbiota in the giant panda due to its unique bamboo-specialized diet, using both culture-dependent and culture-independent approaches. Both approaches have revealed the existence of bacterial strains and metabolomic capacities consistent with cellulose metabolism [11], [17]. Similar to giant pandas, red pandas are also bamboo specialists. However, little is known about the gut microbiota in this species. In this study we characterized the microbiota in fecal samples collected from both wild and captive red pandas using next-generation deep sequencing.

Interestingly, we observed distinct bacterial communities between wild and captive red pandas based on both membership and structure. The wild red pandas had significantly higher community diversity, richness and evenness than the captive red pandas. In addition, the variation of community structures in wild red pandas was also significantly higher than that in captive red pandas.

Diet has been considered as a major driver shaping mammalian gut microbial community structures [6], [18], [19], [20]. In a longitudinal study, Williams *et al.* reported that giant pandas altered their bamboo consumption preferences in a 14-month period. Interestingly, significant linear and quadratic relationships between *lactobacilli* and *Bacteroides* spp. and leaf consumption behavior were also observed, indicating seasonal changes in diet preference might affect bacterial communities in the gut of giant pandas [21]. We thus speculated that the differences in community structure between the two groups of red pandas were due to their different diets. The diet of the captive red panda is well controlled. In addition to bamboo, captive red pandas are also fed with a corn bun (30 g), apple (200 g), banana (200 g), and flour paste (50 g) composed of corn, peanut and soybean. On the contrary, wild red pandas rely on bamboo and whatever else is available in the environment. A variety of foods have been discovered in wild red panda feces including small mammals, birds, eggs, flowers and berries [10]. It is very likely that the heterogeneity of dietary patterns in wild red pandas is the major cause of the bigger variation in wild red panda gut bacterial communities and drives the divergence of wild and captive red pandas.

The effect of diet on the abundance of individual bacterial species has also been demonstrated recently. By using germ-free mice Faith *et al.* assessed the effect of refined diet on changes in the abundance of 10 sequenced human gut bacteria (19). They developed a statistical model that explained >60% of the variation in bacterial abundance caused by diet perturbations and identified factors in the diet that best explained changes in each community member. In our study, the relative abundances of several OTUs (e.g. OTU001, OTU003) differed significantly between the wild and captive red pandas. However, their roles in red pandas' health and disease and the factors that lead to these different OTUs remain unknown. Future longitudinal studies with larger sample size and refined diet are desired to decipher the rules governing the composition and structure of the gut microbiota in red pandas.

Another interesting discovery in our study is that, compared to other mammals, red pandas harbor relatively less diverse bacterial communities, which is unlikely due to the sequencing depth because the Good's coverage of each bacterial community was >97%. The low bacterial diversity might be caused by other possible factors such as phylogeny, biogeography and very likely diet (bamboo) due to its highly fibrous nature and antibacterial activities [22].

Given their unique bamboo-specialized dietary patterns, we also expected to detect cellulose degraders from red pandas. Interestingly, a total of 10 OTUs among the top 50 OTUs identified from red pandas were related to known cellulose degraders, and 16 OTUs matched those from giant pandas [11]. These data suggest that the gut microbiota in red pandas might also play important roles in the digestion of bamboo. Of note, phylogenetic analysis based solely on the 16S rRNA gene indicated the presence of cellulose degraders. Future experiments such as culture-based approaches to screen bacterial isolates and metagenomics approaches to identify cellulase genes and cellulose degradation pathways are desired to identify and characterize the cellulose degraders.

In conclusion, this study provides the first characterization of the gut microbiota in the red panda using next-generation sequencing techniques. We observed that the wild and captive red panda harbor distinct bacterial communities. Furthermore, phylogenetic analysis showed that a considerable number of OTUs were related to cellulose degraders. Our study gives insight into the composition and structure of the gut microbiota in red pandas and paves the way for future investigations into how to better manipulate diets and how to better manage gastrointestinal tract disorders in captive red pandas.

## Materials and Methods

### Ethics Statement

Before sample collection, all animal work was approved by the Institutional Animal Care and Use Committee of the Sichuan Agricultural University under permit number DKY-B20130302 and Fengtongzhai National Reserve for non-invasive sample collection of feces from the red panda under permit number SLH[2012]695.

### Sample collection

Fecal samples were collected from captive and wild red pandas, and immediately put into a liquid nitrogen container and stored at −80°C. Sixteen fecal samples of wild animals were collected from Fengtongzhai National Nature Reserve (Baoxing, Sichuan Province, China) with the help of experienced trackers. Six samples from captive red pandas were obtained from Bifengxia Ecological Zoo (Ya'an, Sichuan Province, China).

### DNA extraction and pyrosequencing

DNA was extracted from the inner part of the frozen fecal samples (0.25 g) using the MO BIO PowerFecal™ DNA Isolation Kit (MO BIO Laboratories, Carlsbad, CA, USA) according to the manufacturer's protocol. The DNA concentration was measured by Nanodrop (Thermo Scientific). DNA pyrosequencing was performed by Beijing Genomics Institute (BGI Shenzhen, China) via 454 Life Sciences/Roche GS FLX Titanium instrument. Briefly, the V1–V3 hypervariable regions of the bacterial 16S rRNA gene were amplified from extracted DNA using bar-coded primers (forward: CCGTCAATTCMTTTGAGTTT, reverse: ACTCCTACGGGAGGCAGCAG). Each 50 µl PCR reaction contained 50 ng DNA, 41 µl molecular biology grade water, 5 µl 10 × FastStart High Fidelity Reaction Buffer with 18 mM MgCl_2_, 1 µl dNTPs (10 mM each), 1 µl Fusion Primer A (10 µM), 1 µl Fusion Primer B (10 µM), and 1 µl FastStart High Fidelity Enzyme Blend (5 U/µl). PCR was performed at 95°C for 2 min; followed by 30 cycles of 95°C for 20 s, 50°C for 30 s, and 72°C for 5 min; followed by a final extension at 72°C for 10 min.

The resulting amplicons were purified using Agencourt AMPure beads. The quality and quantity of the amplicon libraries were assessed by the BioAnalyzer DNA 1000 LabChip (Agilent, Palo Alto, CA, USA). Each amplicon library was then diluted to include 1×10^9^ molecules per µl before being pooled with equal volume. The pooled amplicon libraries were then diluted to 1×10^7^ molecules/µl. Emulsion PCR and sequencing were performed according to the manufacturer's instructions.

### Sequence analysis

Pyrosequencing reads were processed and analyzed using mothur v1.31 [23] following the 454 SOP on the mothur wiki (http://www.mothur.org/wiki/454_SOP) and Schloss *et al.* 2011 [23]. The raw sff file was first denoised by using the PyroNoise algorithm [24] implemented in mothur as the shhh.flows command. Chimeric sequences were removed using the Uchime algorithm [25]. A preclustering methodology [26] was used to further reduce sequencing noise. Sequences that had a length of at least 200 bp and passed the sequencing error reducing and chimera detection and removal steps were considered high quality sequences and were then assigned to OTUs using an average neighbor algorithm with a 97% similarity cutoff. OTUs were classified at the genus level using the Bayesian method [27]. The number of reads per sample was randomly subsampled to 1300 to reduce bias caused by sequencing effort before downstream alpha and beta diversity analyses.

### Ecological and statistical analyses

Good's coverage, rarefaction curve analyses and alpha diversities including community diversity (Inverse Simpson [28] and Shannon index [29]), richness (observed number of OTUs) and evenness (Shannon evenness) were calculated using mothur. Beta diversity measurements, including Jaccard [30] and ThetaYC [14] distances, were calculated to determine the dissimilarity between the communities' membership and structure, respectively.

The Mann Whitney test was used to test the differences in alpha diversities (Shannon Diversity index, inverse Simpson index, richness and evenness) between the wild and captive red pandas. The Kruskal-Wallis test with Dunn's correction was performed to examine the differences in the between- and within-group pairwise ThetaYC distances. A P<0.05 was considered statistically significant. Linear discriminant analysis effect size (LEfSe) [15], which takes into account both statistical significance and biological relevance, was conducted to identify OTUs differentially represented between wild and captive red pandas.

### Phylogenetic tree construction

The 16S rRNA genes of 71 cellulolytic bacteria from GenBank (accession numbers shown in supplementary table S2) and the 85 OTUs reported by Zhu et al (Table S3) [11] were downloaded. By combining these sequences with the top 50 most abundant OTUs detected in our data, which accounted for 97% of the total number of clean sequences, we constructed a neighbor-joining tree based on the Kimura 2-parameter model [16] of the 16S rDNA using MEGA 5 [31], with a bootstrap of 1000 replicates performed to evaluate the reliability of the tree.

## Supporting Information

Figure S1 **Rarefaction curve analyses of bacterial community richness as a function of sequencing depth.** Bacterial communities collected from wild and captive red pandas are presented as yellow circles and green squares, respectively.
(EPS)

Table S1 **Number of sequences and Good's coverage.**
(DOCX)

Table S2 **16S rRNA sequences of cellulolytic bacterial species.**
(DOC)

Table S3 **The microbial flora of wild and captive pandas downloaded from Zhu et al.**
(DOC)
