# Supplementary material for: Characterization of the Gut Microbiota in the Red Panda (Ailurus fulgens)
Source: PLoS One. 2014 Feb 3;9(2):e87885. doi: 10.1371/journal.pone.0087885 (PMC3912123; doi:10.1371/journal.pone.0087885)
Supplement: Table S1 — Number of sequences and Good's coverage. (DOCX) [file pone.0087885.s002.docx]

Table S1 Number of sequences and Good’s coverage

| Sample Label | Source | # Sequences | Good's Coverage Before Subsampling | Good's Coverage  After Subsampling |
| --- | --- | --- | --- | --- |
| 34 | Wild Red Panda | 3002 | 0.991 | 0.982 |
| 35 | Wild Red Panda | 1365 | 0.990 | 0.990 |
| 36 | Wild Red Panda | 2872 | 0.989 | 0.975 |
| 37 | Wild Red Panda | 2075 | 0.980 | 0.973 |
| 38 | Wild Red Panda | 2506 | 0.990 | 0.985 |
| 39 | Wild Red Panda | 1883 | 0.987 | 0.984 |
| 40 | Wild Red Panda | 5170 | 0.997 | 0.99 |
| 41 | Wild Red Panda | 2904 | 0.990 | 0.985 |
| 42 | Wild Red Panda | 1987 | 0.972 | 0.965 |
| 43 | Wild Red Panda | 3215 | 0.994 | 0.986 |
| 44 | Wild Red Panda | 5929 | 0.994 | 0.988 |
| 45 | Wild Red Panda | 3668 | 0.996 | 0.987 |
| 46 | Wild Red Panda | 3440 | 0.998 | 0.993 |
| 47 | Wild Red Panda | 2444 | 0.990 | 0.979 |
| 48 | Wild Red Panda | 1951 | 0.993 | 0.993 |
| 49 | Wild Red Panda | 3759 | 0.992 | 0.984 |
| 66 | Captive Red Panda | 3038 | 0.999 | 0.998 |
| 67 | Captive Red Panda | 2895 | 0.994 | 0.991 |
| 68 | Captive Red Panda | 2462 | 0.988 | 0.982 |
| 69 | Captive Red Panda | 1656 | 0.990 | 0.989 |
| 70 | Captive Red Panda | 3234 | 0.996 | 0.993 |
| 71 | Captive Red Panda | 2167 | 0.991 | 0.988 |
