# Supplementary material for: Characterization of the Gut Microbiota in the Red Panda (Ailurus fulgens)
Source: PLoS One. 2014 Feb 3;9(2):e87885. doi: 10.1371/journal.pone.0087885 (PMC3912123; doi:10.1371/journal.pone.0087885)
Supplement: Table S2 — 16S rRNA sequences of cellulolytic bacterial species. (DOC) [file pone.0087885.s003.doc]

**Table S2 16S rRNA sequences of cellulolytic bacterial species**

| Phylogeny | Genus | Species | GenBank ID |
| --- | --- | --- | --- |
| Phylum Firmicutes, Class Clostridia, Order Clostridiales | | | |
| Fam. Syntrophomonodaceae | *Caldocellulosiruptor* | *saccharolyticus* | NR_036878.1 |
|  | *Caldocellulosiruptor* | *lactoaceticus* | NR_026231.1 |
|  | *Caldocellulosiruptor* | *kristjanssonii* | NR_025279.1 |
|  | *"Anaerocellum"* | *thermophilum* | L09180.1 |
| Fam. Lachnospiraceae | *Butyrivibrio* | *fibrisolvens* | EF427364.1 |
|  | *Ruminococcus* | *flavefaciens* | AM748742.1 |
|  | *Ruminococcus* | *succinogenes* | M62696.1 |
|  | *Ruminococcus* | *albus* | AF079847.1 |
| Fam. Clostridiaceae | *Clostridium* | *acetobutylicum* | AB675523.1 |
|  | *Clostridium* | *chartatabidum* | NR_029239.1 |
|  | *Clostridium* | *cellulovorans* | AF132735.2 |
|  | *Clostridium* | *herbivorans* | L34418.1 |
|  | *Clostridium* | *cellulosi* | NR_044624.1 |
|  | *Clostridium* | *cellobioparum* | NR_026104.1 |
|  | *Clostridium* | *papyrosolvens* | NR_026102.1 |
|  | *Clostridium* | *josui* | AB011057.1 |
|  | *Clostridium* | *cellulolyticum* | NR_102768.1 |
|  | *Clostridium* | *Aldrichii P-1* | NR_026099.2 |
|  | *Clostridium* | *stercorarium* | L09174.1 |
|  | *Clostridium* | *Thermocellum ATCC 27405* | NR_074629.1 |
|  | *Clostridium* | *thermocellum CS8* | JX912712.1 |
|  | *Clostridium* | *thermocellum (DSM 1237)* | L09173.1 |
|  | *Clostridium* | *cellulofermentans DSM 5628* | NR_026100.1 |
|  | *Clostridium* | *Thermocopriae JT-3* | NR_025898.1 |
|  | *Clostridium* | *sp. C7* | HM240780.1 |
|  | *Bacteroides* | *sp. P-1* | JX041639.1 |
|  | *Bacteroides* | *cellulosolvens* | L35517.1 |
|  | *Acetivibrio* | *cellulolyticus* | L35516.1 |
|  | *Acetivibrio* | *Cellulosolvens CD2* | NR_025917.1 |
|  | *Acetivibrio* | *Cellulosolvens ATCC 35928* | L35515.1 |
|  | *Acetivibrio* | *Cellulosolvens ATCC 33288* | L35516.1 |
| Phylum Firmicrtes, Class Bacilli, Order Bacillales | | | |
| Fam. Thermoactinomyceta | *Thermoactinomyces* | *sp. YX* | DQ225173.1 |
|  | *Caldibacillus* | *cellulovorans* | AF163837.1 |
| Fam. Bacillaceae | *Bacillus* | *circulans* | NR_042726.1 |
| Phylum Actinobacteria, Order Actinomycetales | | | |
| Subord. Frankineae, Fam. Acidothermaceae | *Acidothermus* | *cellulolyticus* | AJ007290.1 |
|  | *Cellulomonas* | *biazotea* | X83802.1 |
| Subord. Micromonosporineae, Fam. Cellulomonadaceae | *Cellulomonas* | *cellasea* | X83804.1 |
|  | *Cellulomonas* | *cellulans* | X83809.1 |
|  | *Cellulomonas* | *fimi* | NR_074509.1 |
|  | *Cellulomonas* | *flavigena* | X83799.1 |
|  | *Cellulomonas* | *gelida* | X83800.1 |
|  | *Cellulomonas* | *iranensis* | NR_024914.1 |
|  | *Cellulomonas* | *persica* | AF064701.1 |
|  | *Cellulomonas* | *uda* | X83801.1 |
| Fam. Micromonosporaceae | *Streptomyces* | *reticuli* | GU383165.1 |
| Subordere Streptomycineae,  Fam. Streptomycetaceae | *Streptomyces* | *aureofaciens* | EF017714.1 |
|  | *Streptomyces* | *flavogriseus* | NR_074559.1 |
|  | *Streptomyces* | *lividans* | X86354.1 |
|  | *Streptomyces* | *nitrosporeus* | HQ439420.1 |
|  | *Streptomyces* | *rochei* | KC522303.1 |
|  | *Streptomyces* | *thermovulgaris* | Z68094.1 |
| Subord. Streptosporangiaceae,  Fam. Nocardiopsaceae | *Thermobifida* | *alba* | AB304877.1 |
|  | *Thermobifida (Thermomonospora)* | *fusca* | AM932257.1 |
|  | *Thermobifida* | *cellulolytica* | AJ298059.1 |
|  | *Thermomonospora* | *curvata* | X97893.1 |
|  | *Microbispora* | *bispora JCM3082* | U58524.1 |
|  | *Microbispora* | *Bispora ATCC19993* | U58523.1 |
| Phylum Fibrobacteres, Class Fibrobacteres, Order Fibrobacterales | | | |
| Fam. Fibrobacteriaceae | *Fibrobacter* | *Succinogenes S85* | AJ496032.1 |
| Phylum Bacteroidetes, Class Sphingobacteria, Order Sphingobacteriales | | | |
| Fam. Flexibacteriaceae | *Sporocytophaga* | *Myxococcoides DSM 11118* | NR_025463.1 |
|  | *Sporocytophaga* | *myxococcoides* | AB681028.1 |
|  | *Cytophaga* | *sp.* | U63940.1 |
| Phylum Bacteroidetes, Class Flavobacteria, Order Flavobacteriales | | | |
| Fam. Flavobacteriaceae | *Flavobacterium* | *johnsoniae* | NR_044738.1 |
| Phylum Proteobacteria, Class Betaproteobacteria, Order Burkholderiales | | | |
| Fam. Alcaligenaceae | *Achromobacter* | *piechaudii* | AF237784.1 |
| Phylum Proteobacteria, Class Gammaproteobacteria, Order Xanthomonadales | | | |
| Fam. Xanthomonadaceae | *Xanthomonas* | *sp.* | AY271755.1 |
| Phylum Proteobacteria, Class Gammaproteobacteria, Order Pseudomonadales | | | |
| Fam. Pseudomonaceae | *Cellvibrio* | *vulgaris* | NR_025209.1 |
|  | *Cellvibrio* | *fulvus* | NR_025210.1 |
|  | *Cellvibrio* | *gilvus* | NR_074443.1 |
|  | *Cellvibrio* | *mixtus* | KC329916.1 |
|  | *Pseudomonas* | *fluorescens (cellulosa)* | DQ282185.1 |
|  | *Pseudomonas* | *mendocina* | M59154.1 |
| Phylum Proteobacteria, Class Deltaproteobacteria | | | |
| Fam. unclassified | *Myxobacter* | *sp. AL-1* | FJ544850.1 |
