# Supplementary material for: Characterization of the Gut Microbiota in the Red Panda (Ailurus fulgens)
Source: PLoS One. 2014 Feb 3;9(2):e87885. doi: 10.1371/journal.pone.0087885 (PMC3912123; doi:10.1371/journal.pone.0087885)
Supplement: Table S3 — The microbial flora of wild and captive pandas downloaded from Zhu et al. (DOC) [file pone.0087885.s004.doc]

**Table S3 the microbial flora of wild and captive pandas downloaded from Zhu et al.**

|  | Phylum | GenBank ID |
| --- | --- | --- |
| OTU1 Zhu | Proteobacteria | JF920308 |
| OTU2 Zhu | Proteobacteria | JF920309 |
| OTU3 Zhu | Proteobacteria | JF920310 |
| OTU4 Zhu | Proteobacteria | JF920311 |
| OTU5 Zhu | Proteobacteria | JF920312 |
| OTU6 Zhu | Proteobacteria | JF920313 |
| OTU7 Zhu | Proteobacteria | JF920314 |
| OTU8 Zhu | Proteobacteria | JF920315 |
| OTU9 Zhu | Proteobacteria | JF920316 |
| OTU10 Zhu | Proteobacteria | JF920317 |
| OTU11 Zhu | Proteobacteria | JF920318 |
| OTU12 Zhu | Proteobacteria | JF920319 |
| OTU13 Zhu | Firmicutes-Bacilli | JF920320 |
| OTU14 Zhu | Firmicutes-Bacilli | JF920321 |
| OTU15 Zhu | Firmicutes-Bacilli | JF920322 |
| OTU16 Zhu | Firmicutes-Bacilli | JF920323 |
| OTU17 Zhu | Firmicutes-Bacilli | JF920324 |
| OTU18 Zhu | Firmicutes-Bacilli | JF920325 |
| OTU19 Zhu | Firmicutes-Bacilli | JF920326 |
| OTU20 Zhu | Firmicutes-Bacilli | JF920327 |
| OTU21 Zhu | Firmicutes-Bacilli | JF920328 |
| OTU22 Zhu | Firmicutes-Bacilli | JF920329 |
| OTU23 Zhu | Firmicutes-Bacilli | JF920330 |
| OTU24 Zhu | Firmicutes-Bacilli | JF920331 |
| OTU25 Zhu | Firmicutes-Bacilli | JF920332 |
| OTU26 Zhu | Firmicutes-Bacilli | JF920333 |
| OTU27 Zhu | Firmicutes-Bacilli | JF920334 |
| OTU28 Zhu | Firmicutes-Bacilli | JF920335 |
| OTU29 Zhu | Firmicutes-Bacilli | JF920336 |
| OTU30 Zhu | Firmicutes-Bacilli | JF920337 |
| OTU31 Zhu | Firmicutes-Bacilli | JF920338 |
| OTU32 Zhu | Firmicutes-Bacilli | JF920339 |
| OTU33 Zhu | Firmicutes-Bacilli | JF920340 |
| OTU34 Zhu | Firmicutes-Bacilli | JF920341 |
| OTU35 Zhu | Firmicutes-Bacilli | JF920342 |
| OTU36 Zhu | Firmicutes-Bacilli | JF920343 |
| OTU37 Zhu | Firmicutes-Bacilli | JF920344 |
| OTU38 Zhu | Firmicutes-Bacilli | JF920345 |
| OTU39 Zhu | Firmicutes-Bacilli | JF920346 |
| OTU40 Zhu | Firmicutes-Bacilli | JF920347 |
| OTU41 Zhu | Firmicutes-Bacilli | JF920348 |
| OTU42 Zhu | Firmicutes-Clostridia | JF920349 |
| OTU43 Zhu | Firmicutes-Clostridia | JF920350 |
| OTU44 Zhu | Firmicutes-Clostridia | JF920351 |
| OTU45 Zhu | Firmicutes-Clostridia | JF920352 |
| OTU46 Zhu | Firmicutes-Clostridia | JF920353 |
| OTU47 Zhu | Firmicutes-Clostridia | JF920354 |
| OTU48 Zhu | Firmicutes-Clostridia | JF920355 |
| OTU49 Zhu | Firmicutes-Clostridia | JF920356 |
| OTU50 Zhu | Firmicutes-Clostridia | JF920357 |
| OTU51 Zhu | Firmicutes-Clostridia | JF920358 |
| OTU52 Zhu | Firmicutes-Clostridia | JF920359 |
| OTU53 Zhu | Firmicutes-Clostridia | JF920360 |
| OTU54 Zhu | Firmicutes-Clostridia | JF920361 |
| OTU55 Zhu | Firmicutes-Clostridia | JF920362 |
| OTU56 Zhu | Firmicutes-Clostridia | JF920363 |
| OTU57 Zhu | Firmicutes-Clostridia | JF920364 |
| OTU58 Zhu | Firmicutes-Clostridia | JF920365 |
| OTU59 Zhu | Firmicutes-Clostridia | JF920366 |
| OTU60 Zhu | Firmicutes-Clostridia | JF920367 |
| OTU61 Zhu | Firmicutes-Clostridia | JF920368 |
| OTU62 Zhu | Firmicutes-Clostridia | JF920369 |
| OTU63 Zhu | Firmicutes-Clostridia | JF920370 |
| OTU64 Zhu | Firmicutes-Clostridia | JF920371 |
| OTU65 Zhu | Firmicutes-Clostridia | JF920372 |
| OTU66 Zhu | Firmicutes-Clostridia | JF920373 |
| OTU67 Zhu | Firmicutes-Clostridia | JF920374 |
| OTU68 Zhu | Firmicutes-Clostridia | JF920375 |
| OTU69 Zhu | Firmicutes-Clostridia | JF920376 |
| OTU70 Zhu | Firmicutes-Clostridia | JF920377 |
| OTU71 Zhu | Firmicutes-Clostridia | JF920378 |
| OTU72 Zhu | Firmicutes-Clostridia | JF920379 |
| OTU73 Zhu | Firmicutes-Clostridia | JF920380 |
| OTU74 Zhu | Firmicutes-Clostridia | JF920381 |
| OTU75 Zhu | Cyanobacteria | JF920382 |
| OTU76 Zhu | Acidobacteria | JF920383 |
| OTU77 Zhu | Acidobacteria | JF920384 |
| OTU78 Zhu | Acidobacteria | JF920385 |
| OTU79 Zhu | Acidobacteria | JF920386 |
| OTU80 Zhu | Acidobacteria | JF920387 |
| OTU81 Zhu | Acidobacteria | JF920388 |
| OTU82 Zhu | Acidobacteria | JF920389 |
| OTU83 Zhu | Acidobacteria | JF920390 |
| OTU84 Zhu | Cyanobacteria | JF920391 |
| OTU85 Zhu | Bacteroidetes | JF920392 |
